# Induction of Salt Tolerance in *Arabidopsis thaliana* by Volatiles From *Bacillus amyloliquefaciens* FZB42 via the Jasmonic Acid Signaling Pathway

**DOI:** 10.3389/fmicb.2020.562934

**Published:** 2020-11-12

**Authors:** Shaofang Liu, Yuan Tian, Mei Jia, Xiang Lu, Liang Yue, Xia Zhao, Weigen Jin, Yun Wang, Yubao Zhang, Zhongkui Xie, Ruoyu Wang

**Affiliations:** ^1^Gaolan Station of Agricultural and Ecological Experiment, Northwest Institute of Eco-Environment and Resources, Chinese Academy of Sciences, Lanzhou, China; ^2^Key Laboratory of Bioprocess Engineering of Jiangxi Province, College of Life Sciences, Jiangxi Science and Technology Normal University, Nanchang, China; ^3^School of Chemistry, Biology, and Materials Science, East China University of Technology, Nanchang, China; ^4^Key Laboratory of Stress Physiology and Ecology in Cold and Arid Regions of Gansu Province, Lanzhou, China; ^5^Key Laboratory of Desert and Desertification, Northwest Institute of Eco-Environment and Resources, Chinese Academy of Sciences, Lanzhou, China

**Keywords:** *Bacillus amyloliquefaciens* FZB42, VOCs, jasmonic acid, salt tolerance, *Arabidopsis*

## Abstract

Previously, we showed that *Bacillus amyloliquefaciens* FZB42 can confer salt tolerance in plants by root inoculation under salt stress condition, and the FZB42 volatile organic compounds (VOCs) promoted plant growth and development under non-salt stress condition. In the present study, we investigated the mechanism that allows FZB42 VOCs to confer salt tolerance in *Arabidopsis* without colonization of plant roots. We found that FZB42 VOCs significantly increased the biomass of *Arabidopsis* and also maintained the leaf chlorophyll content under salt stress condition. Physiological tests showed that the plant anti-oxidation system was activated by FZB42 VOCs, where higher peroxidase (POD), catalase (CAT), and superoxide dismutase (SOD) activities were detected in plants exposed to FZB42 VOCs compared with non-exposed plants. In addition, FZB42 VOCs increased the leaf total soluble sugars (TSS) content but decreased the proline content compared with the non-exposed plants. Moreover, FZB42 VOCs significantly decreased the Na^+^ contents of the whole plants and induced the expression of genes (*NHX1*; Na^+^/H^+^ exchanger 1 and *HKT1*; high-affinity K^+^ transporter 1) that function to alleviate Na^+^ toxicity. Furthermore, analysis of mutants with defects in specific hormone pathways showed that FZB42 VOCs induced salt tolerance in plants by modulating jasmonic acid (JA) signaling, which was confirmed by the up-regulation of JA synthesis, defense-related genes, and JA biosynthesis inhibitor tests. The results of this study provide new insights into the molecular mechanism related to the interactions between plant growth-promoting rhizobacteria and plants under salt stress condition.

## Introduction

Plant growth-promoting rhizobacteria (PGPR) are naturally free-living soil microorganisms that colonize plant roots and facilitate plant growth (Jastrow and Miller, 1991; Mayak et al., 1999). Many PGPR have been widely studied and applied to a wide range of agricultural crops for the purpose of growth enhancement, including increased crop yields, plant weight, and seed emergence, due to their ability to form adverse environment-resistant spores (Francis et al., 2010). In addition to their potential effects on plant growth promotion, PGPR play important roles in induced systemic resistance to protect plants against biotic stresses (Yan et al., 2002; Choudhary and Johri, 2009; Rashid et al., 2017; Tahir et al., 2017) and induced systemic tolerance to many abiotic stresses, especially salinity and drought stresses (Kaymak et al., 2009; Yang et al., 2009; Paul and Lade, 2014; Lu et al., 2018; Brilli et al., 2019).

Salinity stress inhibits plant growth and decreases agricultural production, but it also affects the physicochemical properties and ecological balance of soil (Shabala and Cuin, 2007; Shrivastava and Kumar, 2015). Thus, salinity is a critical problem for agriculture throughout the world and it has been investigated widely in the last decade (Mayak et al., 2004; Munns and Tester, 2008; Paul and Lade, 2014). Various strategies have been employed to mitigate this problem. In particular, the development of salt-tolerant breeds is an efficient strategy, but it is time consuming, expensive, seed-specific, and it may cause possible environmental risks (Sergeeva et al., 2006; Hu et al., 2012). Thus, simple and inexpensive biological methods need to be developed. The application of PGPR is an effective approach for improving salt tolerance in order to reduce the agricultural losses caused by salt stress according to previous researches. For example, under salt stress condition, inoculating *Enterobacter* sp. EJ01 into pots containing *Arabidopsis* (Col-0) and tomato (*Lycopersicon esculentum* var. Mill) increased the plant height and biomass (Kim et al., 2014). Halotolerant PGPR such as *Brachybacterium saurashtrense*, *Brevibacterium casei*, and *Haererohalobacter* increased the biomass of *Arachis hypogaea* in the presence of 100 mM NaCl (Shukla et al., 2012). Moreover, inoculating the rhizosphere with *Bacillus amyloliquefaciens* alleviated salt stress and allowed plants such as *Arabidopsis* (Col-0), maize (Jingtian), and rice (*Oryza sativa* L. *indica* var. Narayan), to maintain better growth under salt stress condition (Nautiyal et al., 2013; Chen L. et al., 2016; Liu et al., 2017). The application of PGPR is a simple and economic option for reducing agricultural losses in saline land due to the capacity of PGPR to improve the tolerance of salt by crops (Yildirim et al., 2008), but this effect requires the colonization of PGPR on plants.

Interestingly, in addition to colonization of plant roots, increasing evidence suggests that VOCs released by PGPR also can effectively promote growth and induce salt tolerance in plants (Ryu et al., 2003; Zhang et al., 2008; Bhattacharyya et al., 2015; Hao et al., 2016). It was shown that the VOCs produced by *Bacillus subtilis* GB03 induced salt tolerance in *Arabidopsis* (Col-0) via the tissue-specific regulation of the potassium transporter *HKT1* in different tissues, thereby resulting in lower Na^+^ accumulation throughout the whole plant (Zhang et al., 2008). Moreover, the exposure of *Arabidopsis* (Col-0) to VOCs from *Alcaligenes faecalis* JBCS1294 under salt stress increased the shoot and root length, lateral root number, and fresh weight (Bhattacharyya et al., 2015; Bhattacharyya and Lee, 2017). However, few reports have investigated the molecular mechanism responsible for salt tolerance in plants via the VOCs emitted by PGPR.

*Bacillus amyloliquefaciens* is considered to be a typical PGPR (Idris et al., 2004, 2007) and it is a major plant biostimulant and biocontrol agent (Koumoutsi et al., 2004; Idris et al., 2007; Chen et al., 2009). In a previous work, we found that FZB42, a typical representative strain of *Bacillus amyloliquefaciens*, conferred salt tolerance in *Arabidopsis* by colonizing its roots in a PGPR–plant interaction, through up-regulating plant JA/ethylene (ET) pathways (Liu et al., 2017). Furthermore, we found that the VOCs released by FZB42 promoted plant growth under non-salt stress condition (Hao et al., 2016). So we speculated that FZB42 VOCs might also induce plant salt tolerance. Our pre-experiment results showed that the biomass of plants exposed to FZB42 VOCs were notably higher than that of non-exposed plants under salt stress condition, which were consistent with our speculation. However, it is still unclear which hormonal pathways contribute to this process.

In this study, we investigated the physiological changes of plants exposed to FZB42 VOCs, which might play important roles in the induction of salt tolerance in *Arabidopsis*. We also tested the hormonal pathways using several *Arabidopsis* mutant lines. Furthermore, the expression profiles of genes related to plant growth and stress tolerance in *Arabidopsis* were determined by quantitative reverse transcription PCR (qRT-PCR), specially those involved with photosynthesis, Na^+^ extrusion, abscisic acid (ABA) synthesis, JA synthesis, and JA-mediated defense responses, under salt stress condition with or without exposure to FZB42 VOCs. In addition, the growth phenotypes of *Arabidopsis* grown in Murashige and Skoog (MS) medium with sodium diethyldithiocarbamate (DIECA), a JA biosynthesis inhibitor, were determined under non-salt and salt stress conditions with or without exposure to FZB42 VOCs.

## Materials and Methods

### Bacterial Cultures

*Bacillus amyloliquefaciens* FZB42 was used in this study. FZB42 [deposited as strain 10A6 in the culture collection at the Bacillus Genetic Stock Center (BGSC)] is a Gram-positive, plant-associated bacterium, which can stimulate plant growth (Chen et al., 2007). For routine growth, FZB42 was grown overnight in Luria–Bertani liquid medium with shaking at 200 rpm at 37°C. Cells were obtained by centrifugation at 10000 × *g* for 6 min and re-suspended in sterile water to yield 10^8^ CFU mL^–1^ for use as an inoculum. The non-growth-promoting strain *Escherichia coli* DH5α (TaKaRa, China) was used as a negative control and the culture conditions were the same to those used for FZB42.

### Plant Materials and Treatments

*Arabidopsis thaliana* ecotype Columbia-0 (Col-0) and its mutant lines (donated by Nicole K. Clay) comprising *etr1-3* (ET-insensitive mutant), *eto1* (ET-overproducing mutant), *abi4-102* (ABA-insensitive mutant), *cre1-2* (cytokinin receptor mutant), *aux1-7* (auxin influx mutant), *axr1-12* (auxin-resistant mutant), *ga1* (gibberellin-deficient mutant), *jar1-1* (JA-insensitive mutant), *myc2* (JA-response mutant), *nahG* (salicylic acid-deficient mutant), and *npr1-1* (salicylic acid-response mutant) were used in the present study (Table 1). The seeds were surface sterilized with 2% NaClO for 4 min, followed by washed five to six times with sterile water. After surface disinfection, the seeds were sown on one side of I-plates (Fisher Scientific, Pittsburgh, United States) containing MS medium supplemented with 0 or 100 mM NaCl. Then, vernalized for 2 days at 4°C in the absence of light.

**TABLE 1:** *Arabidopsis* mutants used in this study.

Mutants	Stock name	Locus	Function
*etr1-3*	CS3070	AT1G66340	Ethylene-insensitive mutant
*eto1*	CS3072	AT3G51770	Ethylene-overproducing mutant
*abi4-102*	CS3837	AT2G40220	ABA-insensitive mutant
*cre1-2*	CS6563	AT3G54340	Cytokinin receptor mutant
*aux1-7*	CS9583	AT2G38120	Auxin polar transport- deficient mutant
*axr1-12*	CS3076	AT1G05180	Auxin-resistant mutant
*ga1-3*	CS3104	AT4G02780	Gibberellin-deficient mutant
*jar1-1*	CS8072	AT2G46370	Jasmonate insensitive mutant
*myc2*	SALK_017005	AT1G32640	JA-response mutant
*nahG*	CS67803	AT5G33340	Salicylic acid-deficient mutant
*npr1-1*	CS3726	AT1G64280	Salicylic acid-response mutant

After vernalization, the seeds were inoculated with 20 μL of FZB42/DH5α suspension culture or sterile water, which was dropped on the other side of the I-plates that did not contain seeds. Plates were sealed with parafilm and placed in growth cabinets set to 22°C with a 16/8 h light/dark photoperiod. After exposure to the VOCs for 15 and 20 days, the shoot and roots were collected from Col-0 seedlings for physiological analyses, and the mutants were collected at 20 days after exposure to VOCs to determine their physiological parameters. All of the experiments were repeated three times as three biological replicates and each replicate included 120 plants.

### JA Inhibition Assay

DIECA (Real-Times, China) was used as a JA inhibitors because it inhibits lipoxygenase (LOX), which is a key enzyme during JA biosynthesis (Chen X. et al., 2016). *Arabidopsis* Col-0 seeds were sown on MS medium containing DIECA with or without 100 mM NaCl on the I-plates. After vernalization, the seeds were inoculated with 20 μL of FZB42 suspension culture or sterile water, which was dropped on the other side of the I-plates as described above. The growth performance of plants was determined after exposure to FZB42 VOCs for 20 days.

### Leaf Chlorophyll

The chlorophyll a, b, and a+b contents were determined at 15 and 20 days after exposure to FZB42 VOCs using the method described by Ashraf and Iram (2005), where 0.2 g of fresh leaf tissues was extracted overnight with 80% acetone at 4°C. The solution was obtained after centrifugation at 14000 × *g* for 5 min and the absorbance of the supernatant was measured at 663 nm and 645 nm to detect the chlorophyll a, chlorophyll b, and chlorophyll a+b contents.

### Determination of POD, CAT, and SOD Activities

The POD, SOD, and CAT activities in *Arabidopsis* leaves were assayed according to the method described by Qiu et al. (2007). Each sample comprising 0.2 g of fresh shoot tissues was homogenized in 2 mL of 50 mM ice-cold phosphate buffer (pH 7.8) containing 1 mM EDTA. The homogenate was centrifuged at 15000 × *g* for 15 min at 4°C. The supernatant comprised an enzyme extract containing POD, SOD, and CAT. The POD activity was determined based on the oxidation of guaiacol using hydrogen peroxide. The CAT activity was determined based on the decrease in the level of H_2_O_2_. The SOD activity was measured based on its effectiveness at inhibiting the photoreduction of nitro blue tetrazolium.

### Measurement of TSS and Proline Contents

The TSS contents of the Col-0 seedlings were determined with anthrone reagent as described previously (Irigoyen et al., 1992). First, 500 mg of leaf tissues were crushed in 5 mL of 95% (v/v) ethanol and the insoluble fraction was washed twice with 70% ethanol. After centrifugation at 3500 rpm for 10 min, 0.1 mL of the supernatant was mixed with 3 mL of freshly prepared anthrone and then reacted in a boiling water bath for 10 min. The absorbance of the mixture was read at 625 nm.

The free proline contents in the shoot tissues were quantified as described previously (Bates et al., 1973), where 100 mg of shoot tissues were homogenized in 3% aqueous sulfosalicylic acid. Next, 2 mL of the filtrate was reacted with 2 mL acid-ninhydrin and 2 mL of glacial acetic acid at 100°C for 1 h. The reaction mixture was extracted with 4 mL of toluene for 15–20 s. The optical density of the mixture was measured at 520 nm.

### Determination of Ion Contents

The shoots and roots were harvested from Col-0 seedlings exposed to FZB42 or water for 15 and 20 days, respectively. The K^+^ and Na^+^ contents were determined by inductively coupled plasma-optical emission spectrometry (ICP-OES, PerkinElmer Optima 8000, United States) according to the method described by Zhang et al. (2008).

### Transcript Analyses by qRT-PCR

The expression patterns of genes related to photosynthesis (*TPPH*; trehalose-phosphate phosphatase H and *LHCB4.3*; light harvesting complex photosystem II), Na^+^ extrusion (*NHX1*; Na^+^/H^+^ antiporter and *HKT1*; high-affinity K^+^ transporter 1), ABA synthesis (*NCED3*; 9-cis-epoxycarotenoid dioxygenase), JA synthesis (*LOX4*; lipoxygenase 4), and JA-mediated defense responses (*JMT*; jasmonic acid carboxyl methyltransferase, *PDF1.3*; plant defensin 1.3, *PDF1.2c*; plant defensin 1.2C, and *PDF1.2*; plant defensin 1.2) were analyzed using shoot tissues collected at 15 and 20 days after exposure to FZB42 under both non-salt and salt stress conditions. Total RNA was extracted from shoot tissues using an RNeasy Plant Mini Kit. First-strand cDNA synthesis was got by using a PrimeScript TM RT reagent Kit with gDNA Eraser (Perfect Real Time), which could remove genomic DNA. Quantitative reverse transcription polymerase chain reaction (qRT-PCR) was performed with a Mx3000 P system (Applied Biosystems) using SYBR^®^ Premix Ex Taq^TM^ II (TliRNaseH Plus) (TaKaRa). The *ACT2* gene (GenBank: *AT3G18780*) was used as a quantitative control to normalize the results. The primers used in this study are listed in Supplementary Table S1. The thermal conditions were as follows: 95°C for 30 s, and 40 cycles at 95°C for 5 s and 60°C for 34 s. Each PCR analysis was repeated at least three times. The expression levels of genes were calculated using the threshold cycle2^–ΔΔ*Ct*^ method (Livak and Schmittgen, 2001).

### VOCs Collection and Analysis

The VOCs of FZB42 was collected with solid phase micro extraction (SPME) fiber (Supelco, Bellefonta, PA, United States) according to the method of Tahir et al. (2017). Twenty μL suspension of FZB42 (OD = 2.0) was inoculated into 30 mL of modified MS agar medium in a 200 mL tissue culture vessel. Seven days after incubation, 3 cm SPME fiber SPME fiber was inserted into the headspace of the vessel containing FZB42 and incubated at 50°C for 30 min.

The GC-MS analysis was performed using a Hewlett-Packard 6890 gas chromatograph (GC) coupled to a Hewlett-Packard 5973 mass selective detector (MSD) with a split/splitless injector. The GC was installed with a HP-5 capillary column (30-m; 0.25-mm ID; 0.25-μm film thickness) with the helium was used as carrier gas in constant flow of 1.2 ml/min. Helium was used as the carrier gas at a linear velocity of 28 cm/s, with the injector operating at constant flow of 0.9 ml/min. The GC inlet temperature, which was also the desorption temperature for SPME fiber, was programmed from 80 to 280°C at 4°C/min with initial and final hold times of 1 and 30 min, respectively. The GC initial temperature was maintained at 40°C for 4 min, then ramped to 280°C at a rate of 4°C/min and kept for 10 min. Full mass scan from 1 to 400 amu was acquired in the electron impact ionization mode with electron impact ionization at 70 eV. The MSD was operated with ionization energy of 70 eV, ion source temperature of 230°C and an electron multiplier voltage of 1800 V over the mass range 35–550 Daltons. The mass spectra data for VOC compounds was analyzed using the data in the NIST/EPA/NIH Mass Spectrum Library. Only MS agar medium was used as control. Two separate analyses were performed.

### Role of Acetoin in Plant Growth Under Non-salt and Salt Stress Conditions

*alsD* and *alsS* encoding acetolactate decarboxylase and acetolactate synthase, respectively, are two keys genes involved in acetoin synthesis in *Bacillus*, which play important roles in promoting plant growth (Ryu et al., 2003). *alsD* and *alsS* in FZB42 genome were deleted by the method of Xu et al. (2014). The effect of △*alsD* and △*alsS* on plant growth under non-salt and salt stress conditions were done as the same to the wild strain as shown above. The fresh weight of shoot parts were measured at 17 days after exposure to VOCs.

### Statistical Analysis

Significant differences in the data were determined using analysis of variance (ANOVA) followed by Duncan’s multiple-range tests (*P* < 0.05) using SPSS version 19.0 (SPSS Inc., Chicago, IL, United States) and the *t*-test were used for statistical significance.

## Results

### FZB42 Induced Salt Tolerance in Plants

**FIGURE 1:**
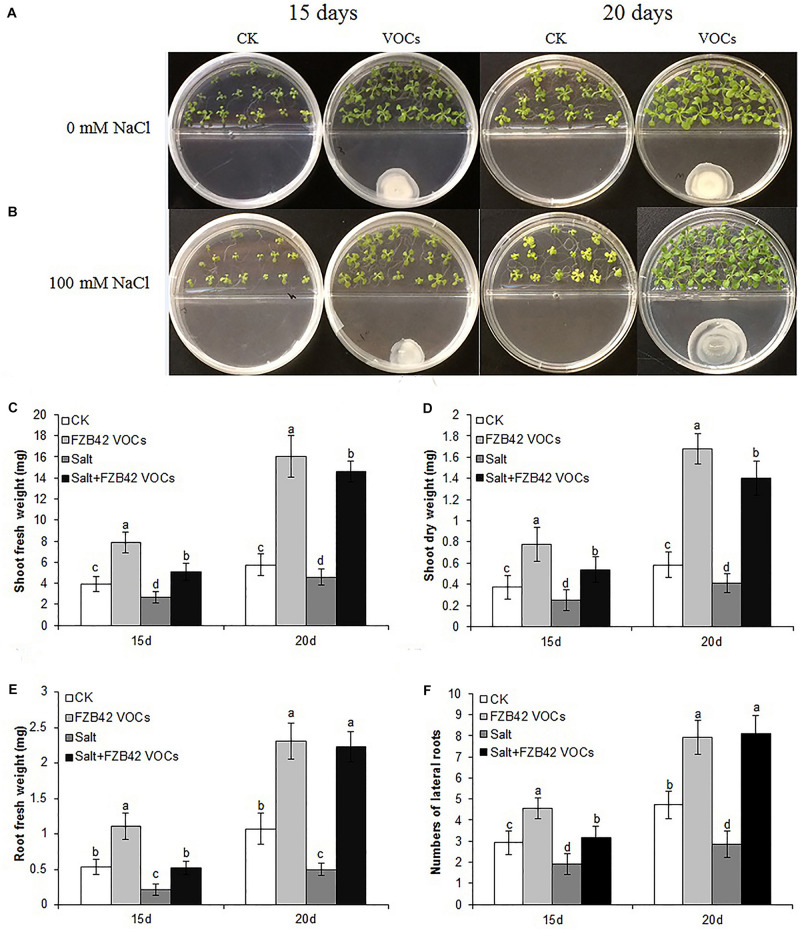
Effects of FZB42 VOCs on growth and salt tolerance in *Arabidopsis*. Representative images of *Arabidopsis* after exposure to VOCs from FZB42 or water for 15 and 20 days, respectively **(A,B)**. Shoot fresh weight **(C)**, shoot dry weight **(D)**, root fresh weight **(E)**, and lateral root number **(F)**. White, light gray, dark gray, and black bars represent CK (only water), FZB42 VOCs (only FZB42 VOCs), Salt (only salt stress), and Salt + FZB42 VOCs (salt stress + FZB42 VOCs) treatments, respectively. Different letters indicate statistically significant differences between treatments (Duncan’s multiple range tests, *P* < 0.05; *n* = 40, mean ± standard deviation).

Under non-salt and salt stress conditions, after exposure to VOCs from FZB42 for 15 and 20 days, *Arabidopsis* (Col-0) plants exhibited robust growth compared with the non-exposed controls (Figures 1A,B) as demonstrated by their enhanced shoot and root biomass (Figures 1C–E). Dramatic differences were also observed in terms of root hair development. The lateral root numbers were significantly higher in *Arabidopsis* plants exposed to FZB42 than the non-exposed plants at both 0 and 100 mM NaCl. However, significant difference of root length was only detect in plants exposed FZB42 VOCs for 20 days (Supplementary Figure S1). These observations clearly demonstrate FZB42 VOCs can promote plant growth and induce plant tolerance to salt stress. The VOCs of *E. coli* strain DH5α could not promote plant growth (Ryu et al., 2003), thus DH5α has been set as a control to exclude the possibility that the growth promotion and tolerance induction come from the increasing carbon dioxide which released when the bacteria growing (Supplementary Figure S2).

### Effects of FZB42 VOCs on Leaf Chlorophyll Contents

**FIGURE 2:**
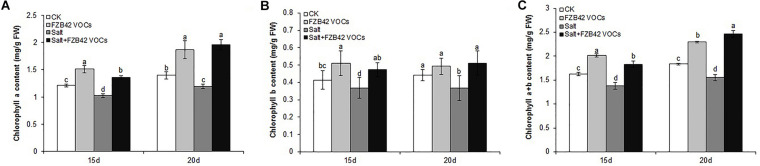
Effects of FZB42 VOCs on chlorophyll contents of *Arabidopsis*. Chlorophyll a (**A**), chlorophyll b (**B**), and chlorophyll a+b (**C**). White, light gray, dark gray, and black bars represent CK (only water), FZB42 VOCs (only FZB42 VOCs), Salt (only salt stress), and Salt+FZB42 VOCs (salt stress+FZB42 VOCs) treatments, respectively. Different letters indicate statistically significant differences between treatments (Duncan’s multiple range tests, *P* < 0.05; *n* = 6, mean ± standard deviation).

**FIGURE 3:**
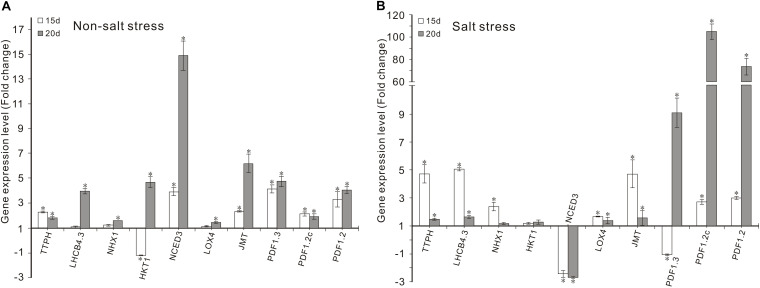
Effects of FZB42 VOCs on the expression of genes related to photosynthesis, Na^+^ balance, ABA and JA synthesis, and JA-mediated defense responses. Expression levels of the genes were detected by qRT-PCR at 15 and 20 days after exposure to FZB42 VOCs at both 0 mM NaCl **(A)** and 100 mM NaCl **(B)**. White and gray bars represent plants exposed to FZB42 for 15 and 20 days, respectively. The X-axis indicates the 10 genes (from left to right: *AT4G39770*, *AT2G40100*, *AT5G27150*, *AT4G10310*, *AT3G14440*, *AT1G72520*, *AT1G19640*, *AT2G26010*, *AT5G44420*, and *AT5G44430*). CK (only water) and Salt (only salt stress) exposed for both 15 and 20 days were selected as calibrator samples, respectively, to detect the effect of FZB42 VOCs on the expression patterns of the ten target genes both under non-salt and salt stress conditions. *Statistically significant differences (*P* < 0.05, *t*-test, *n* = 3, mean ± standard deviation).

In order to examine the impact of FZB42 VOCs on the photosynthetic efficiency of plants under salt stress, we measured the leaf chlorophyll contents and determined the expression level of genes related to photosynthesis (*TPPH* and *LHCB4.3*). Compared with the non-exposed plants, the leaf chlorophyll a, b, and a+b contents in plants exposed to FZB42 VOCs were clearly higher under both non-salt and salt stress, respectively, compared with those in the non-exposed plants (Figure 2). In addition, after exposure to FZB42 for 20 days under salt stress condition, the leaf chlorophyll a, b, and a+b contents in plants increased by 64.7, 38.9, and 58.6%, respectively, compared with non-exposed controls (Figure 2 and Supplementary Table S2), thereby demonstrating that the FZB42 VOCs maintained the plant leaf chlorophyll contents under salt stress condition. These findings were consistent with the much darker green leaves on the *Arabidopsis* plants that were exposed to FZB42 compared with the non-exposed plants (Figures 1A,B). Higher chlorophyll contents in plants exposed to FZB42 VOCs were also detected under non-salt conditions (Figure 2). In addition, the transcriptional levels of *TPPH* and *LHCB4.3* genes were significantly up-regulated by FZB42 VOCs after exposure for 15 and 20 days at 0 and 100 mM NaCl (Figure 3), showing that the VOCs emitted from FZB42 could enhance the efficiency of photosynthesis in plants under non-salt and salt stress conditions.

### Effects of FZB42 VOCs on POD, CAT, and SOD Activities

**FIGURE 4:**
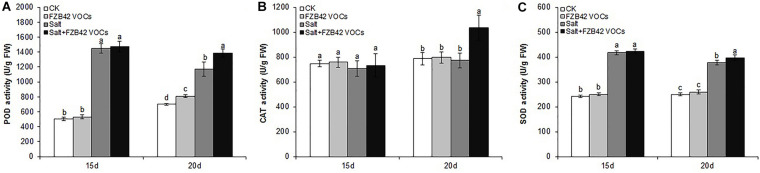
Effects of FZB42 VOCs on the POD, CAT, and SOD activities in *Arabidopsis.* POD activity **(A)**, CAT activity **(B)**, SOD activity **(C)**. White, light gray, dark gray, and black bars represent CK (only water), FZB42 VOCs (only FZB42 VOCs), Salt (only salt stress), and Salt + FZB42 VOCs (salt stress + FZB42 VOCs) treatments, respectively. Different letters indicate statistically significant differences between treatments (Duncan’s multiple range tests, *P* < 0.05; *n* = 6, mean ± standard deviation).

The effects of FZB42 VOCs on POD, CAT, and SOD Activities were detected. We found that FZB42 VOCs did not remarkably increase the POD, CAT, and SOD activities under non-salt stress condition, as no significant difference were detected between VOCs exposed and non-VOCs exposed plants except POD in VOCs exposed plants for 20 days (Figure 4). Under salt stress condition and exposure to FZB42 VOCs for 20 days, the POD, CAT, and SOD activities increased by 18.1, 34.1, and 5.3%, respectively, compared with the non-exposed controls (Figure 4 and Supplementary Table S2). However, no significant differences were detected in the plants after exposure to FZB42 VOCs for 15 days under salt stress condition, although slightly higher antioxidant activities were still obtained. These results suggest that FZB42 VOCs could efficiently alleviate the oxidative stress driven by salt stress to help plants adapt to salt stress, although this response depends on the exposure time.

### Effects of FZB42 VOCs on TSS and Proline Contents

**FIGURE 5:**
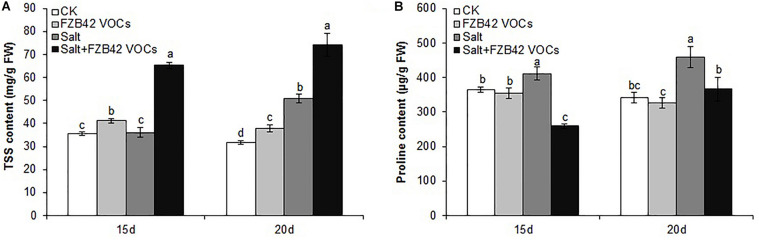
Effects of FZB42 VOCs on osmoprotectants in *Arabidopsis* after treatment for 15 and 20 days. TSS contents **(A)**, proline contents **(B)**. White, light gray, dark gray, and black bars represent CK (only water), FZB42 VOCs (only FZB42 VOCs), Salt (only salt stress), and Salt + FZB42 VOCs (salt stress + FZB42 VOCs) treatments, respectively. Different letters indicate statistically significant differences between treatments (Duncan’s multiple range tests, *P* < 0.05; *n* = 6, mean ± standard deviation).

Under non-salt and salt stress conditions, significantly more TSS accumulated in the plants exposed to FZB42 compared with non-exposed controls after exposure for 15 and 20 days (Figure 5A). Unexpectedly, the changes in the proline contents did not agree with those in the TSS contents. There was no significant difference in the proline contents of plants exposed to FZB42 and non-exposed plants under non-salt stress condition. However, the proline levels were significantly lower in the plants exposed to FZB42 VOCs compared with non-exposed plants after 15 and 20 days under salt stress condition (Figure 5B).

### Effects of FZB42 VOCs on Ion Hemostasis

**FIGURE 6:**
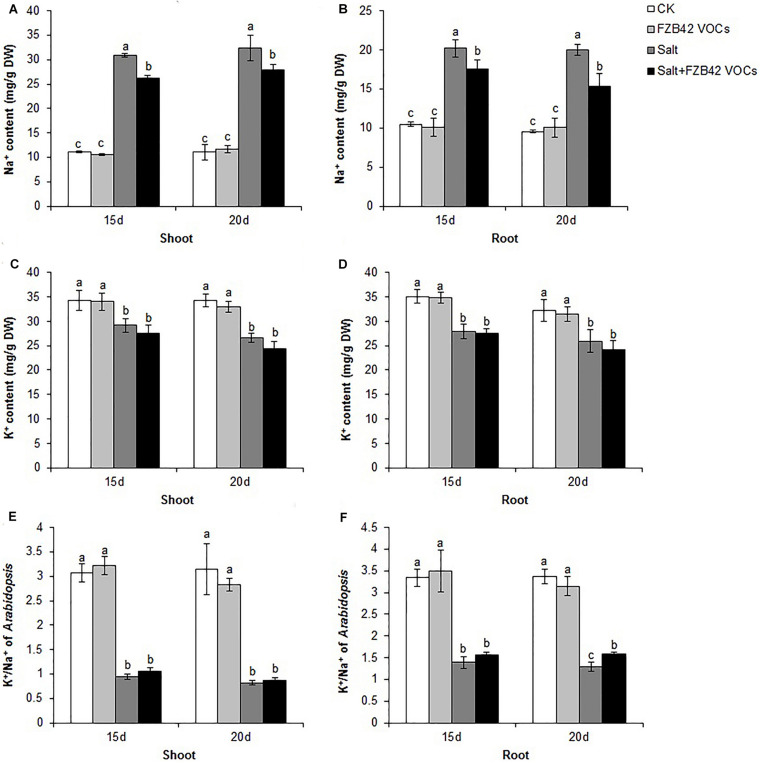
Effects of FZB42 VOCs on the ion contents of *Arabidopsis* after treatment for 15 and 20 days. Na^+^ contents of shoots **(A)** and roots **(B)**. K^+^ contents of shoots **(C)** and roots **(D)**. K^+^/Na^+^ ratio in *Arabidopsis* shoots **(E)** and roots **(F)**. White, light gray, dark gray, and black bars represent CK (only water), FZB42 VOCs (only FZB42 VOCs), Salt (only salt stress), and Salt + FZB42 VOCs (salt stress + FZB42 VOCs) treatments, respectively. Different letters indicate statistically significant differences between treatments (Duncan’s multiple range tests, *P* < 0.05; *n* = 3, mean ± standard deviation).

Under non-salt stress condition, FZB42 VOCs had no effect on the Na^+^ levels of plants compared with non-exposed controls. However, the FZB42 VOCs greatly reduced the Na^+^ contents of plants compared with the non-exposed plants under salt stress condition. At 15 days post-exposure, the Na^+^ contents in the shoots and roots of plants exposed to FZB42 were 84.8 and 87.1% of those in the non-exposed plants, respectively, and at 20 days post-exposure, the Na^+^ contents in the exposed plants were 85.9 and 76.8% of those in non-exposed plants (Figures 6A,B and Supplementary Table S2). There were no significant differences in the K^+^ levels in the plants exposed to FZB42 and the non-exposed plants under non-salt and salt stress conditions (Figures 6C,D), but higher K^+^/Na^+^ ratios were obtained in most of the shoot and root samples from plants exposed to FZB42, but especially in the roots after exposure for 20 days (Figures 6E,F). In agreement with the physiological data, the expression levels of *NHX1* (up 2.4-fold and 1.2-fold after exposure for 15 and 20 days) and *HKT1* (up 1.2-fold and 1.3-fold after exposure for 15 and 20 days), which play important roles in decreasing the Na^+^ contents of plants, were up-regulated by FZB42 VOCs under salt stress condition (Figure 3) thereby indicating that the FZB42 VOCs could effectively reduce the Na^+^ concentration in plants, thus alleviating Na^+^ toxicity.

### Effects of FZB42 VOCs on the Growth of *Arabidopsis* Mutant Lines

**FIGURE 7:**
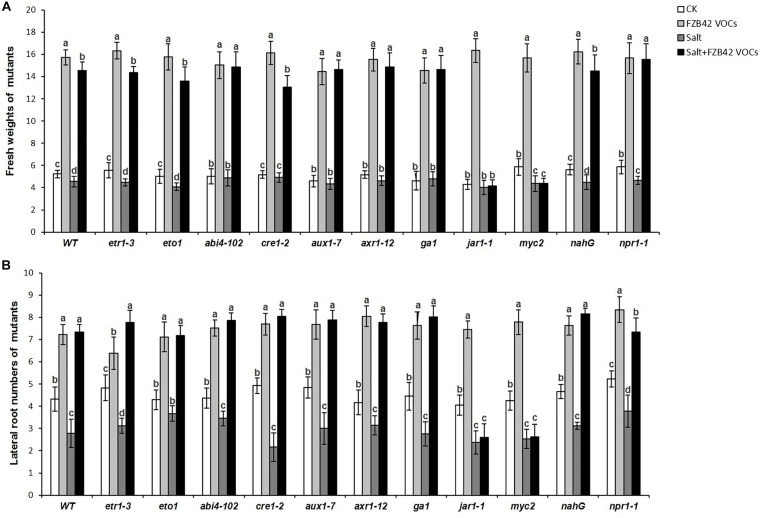
Effects of FZB42 VOCs on shoot growth and salt tolerance of different *Arabidopsis* mutants under non-salt and salt stress conditions. **(A,B)** Indicated fresh weights of shoots and lateral root numbers of the mutants, respectively. White, light gray, dark gray, and black bars represent CK (only water), FZB42 VOCs (only FZB42 VOCs), Salt (only salt stress), and Salt + FZB42 VOCs (salt stress + FZB42 VOCs) treatments, respectively. Different letters indicate statistically significant differences between treatments (Duncan’s multiple range tests, *P* < 0.05; *n* = 30, mean ± standard deviation).

In order to obtain further insights into the roles of phytohormone pathways that might mediate the effects of FZB42 VOCs on salt tolerance induction, 11 *Arabidopsis* mutants (Table 1) comprising *etr1-3*, *eto1*, *abi4-102*, *cre1-2*, *aux1-7*, *axr1-12*, *ga1-3*, *jar1-1*, *myc2*, *nahG*, and *npr1-1* grown under non-salt and salt stress conditions were exposed to FZB42 VOCs. The mutants exposed to FZB42 VOCs had significantly higher fresh weights and lateral root numbers compared with the non-exposed plants under salt stress condition in *etr1-3*, *eto1*, *abi4-102*, *cre1-2*, *ga1*, *aux1-7*, *axr1-12*, *nahG*, and *npr1-1*, however, not in *jar1-1* and *myc2* (Figure 7 and Supplementary Figure S3). Moreover, no notable or lower root length was detected in VOCs exposed *myc2* and *jar1-1* under salt stress condition (Supplementary Figures S4, S5). JAR1 (*AT2G46370*) encodes a jasmonate-amido synthetase, its loss of function mutants (*jar1-1*) are defective in a variety of responses to JA. MYC2 that encodes a MYC-related transcriptional activator regulates diverse JA-dependent functions, its loss of function mutants (*myc2*) is also defective in JA response. The fact that FZB42 VOCs did not significantly promote the growth of *jar1-1* indicated that the induction of salt tolerance conferred by FZB42 VOCs in plants might be related to the JA signaling. And the results that FZB42 VOCs failed to promote help *myc2* tolerate salt stress also confirmed the function of JA signaling in the induced plant salt tolerance by FZB42 VOCs.

Interestingly, the FZB42 VOCs significantly enhanced the growth of all the test mutants under non-salt stress condition according to their fresh weights and root numbers (Figure 7). Overall, the results suggested that FZB42 VOCs could induce salt tolerance in *Arabidopsis* by mainly modulating JA signaling, whereas the plant growth promotion conferred by FZB42 VOCs under non-salt stress condition was not related to JA signaling.

The transcription levels of the key enzyme for JA synthesis (*LOX4*) and four JA-mediated defense response genes (*JMT*, *PDF1.3*, *PDF1.2c*, *PDF1.2*) were up-regulated in *Arabidopsis* after exposure to FZB42 VOCs for 15 and 20 days under non-salt and salt stress conditions. Notable expression level of *PDF1.2c* (105.3-fold) and *PDF1.2* (73.6-fold) was detected in plants exposed FZB42 for 20 days. However, the expression of *NCED3*, a gene involved in ABA synthesis, decreased 2.4-fold and 2.7-fold in plants exposed to FZB42 for 15 and 20 days under salt stress condition, respectively, whereas the expression level of *NCED3* increased 3.9-fold and 14.9-fold under non-salt stress condition. In general, these results indicated that JA might be the key hormone that regulates the defensive response to salt whereas the hormone ABA has little effect.

### Effects of DIECA Treatment on the Growth Phenotype in *Arabidopsis*

**FIGURE 8:**
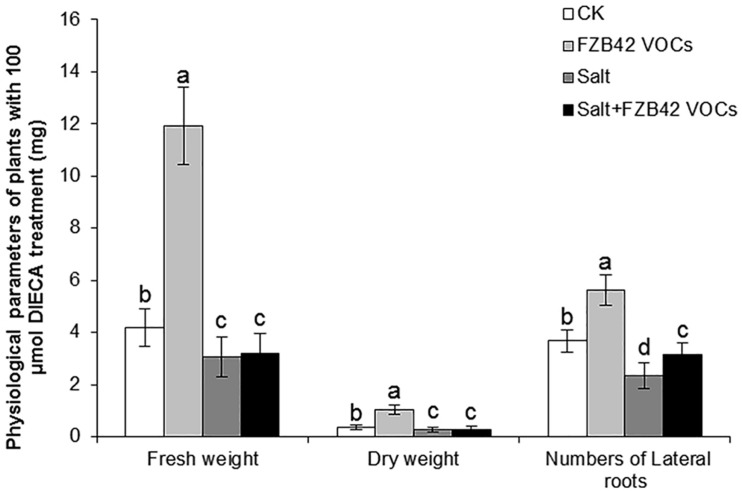
Effects of FZB42 VOCs on growth and salt tolerance in *Arabidopsis* Col-0 under non-salt and salt stress conditions in the presence of JA biosynthesis inhibitor DIECA. White and black bars represent CK (only water), FZB42 VOCs (only FZB42 VOCs), Salt (only salt stress), and Salt + FZB42 VOCs (salt stress + FZB42 VOCs) treatments, respectively. Different letters indicate statistically significant differences between treatments (Duncan’s multiple range tests, *P* < 0.05; *n* = 35, mean ± standard deviation).

The changes in the growth phenotype exhibited by *Arabidopsis* were observed after the application of an inhibitor of JA biosynthesis (DIECA) to further determine the role of JA in salt tolerance. Significantly higher fresh and dry weights of shoots were observed in the plants exposed to FZB42 and treated with DIECA compared with the non-exposed plants under non-salt stress condition, whereas no significant differences were detected in the fresh and dry weights between VOCs exposed plants and non-exposed plants under salt stress condition (Figure 8), thereby indicating that JA signaling pathways had a critical role in the induction of salt tolerance in response to FZB42 VOCs under salt stress condition. However, FZB42 VOCs significantly increased the lateral root numbers of plants treated with DIECA under both non-salt and salt stress conditions (Figure 8).

### GC-MS Analysis of FZB42 VOCs

Volatile organic compounds produced by FZB42 was collected by SPME and GC-MS. Thirty-eight different peaks were identified from FZB42 (Supplementary Figure S6), among which the major peak areas were Pentadecane (ca. 30.26% of the total area of all peaks in the chromatogram, RT 37.63 min) and 3-hydroxy-2-Butanone (acetoin) (ca. 10.94%, RT 3.61 min) (Supplementary Table S3). Compounds Acetic acid (ca. 0.11%, RT 2.38 min) and 3-methyl-2-Butanone (ca. 0.14%, RT 2.33 min) were in relatively low quantities (Supplementary Table S3).

### Effect of **△***alsD* and **△***alsS* on Plant Growth

To confirm the function of acetoin in FZB42 VOCs in plant growth promotion as described by other researchers, *alsD* and *alsS*, key genes involved in acetoin biosynthesis, were deleted, two mutants △*alsD* and △*alsS* that could not produce acetoin were got. Under non-salt stress condition △*alsD* and △*alsS* significantly enhanced the biomass of *Arabidopsis* seedlings like FZB42, and there was no significant difference between the weight of FZB42 and mutants exposed plants (Supplementary Figure S7), which indicated that acetoin had no positive effects on plant growth under non-salt stress condition. Similar results were also observed under salt stress condition, but there was one difference that was the weights of △*alsD* and △*alsS* exposed plants were significantly lower than that of FZB42 exposed plants (Supplementary Figure S7), though the growth curves of FZB42 and these two mutants △*alsD* and △*alsS* were similar (Supplementary Figure S8). These results revealed that acetoin might be the active but not the key component in the induction of salt tolerance in plants.

## Discussion

In our previous study, we showed that the VOCs emitted by FZB42 stimulated the growth and development of plants under non-salt stress condition (Hao et al., 2016). Furthermore, we demonstrated that FZB42 dramatically promoted plant growth under both non-salt and salt stress conditions by root colonization, thereby suggesting that FZB42 could induce salt tolerance in plants via colonizing on the roots of plants (Liu et al., 2017). In this study, we evaluated the growth performance of *Arabidopsis* plants exposed to VOCs from FZB42 under conditions with 0 and 100 mM NaCl but with no direct plant contact. The results indicated that the VOCs emitted from FZB42 effectively induced salt tolerance in *Arabidopsis*, as demonstrated by the significant increases in the shoot/root biomass and lateral root numbers (Figure 1). Therefore, in addition to functioning as an effective biocontrol agent, FZB42 has the potential to facilitate plant growth in saline environments as an environmentally friendly salt tolerance elicitor.

Chlorophyll is vital for plant photosynthesis because it allows plants to absorb energy from light. Under saline conditions, salinity usually decreases the chlorophyll contents of plants (Iqbal et al., 2006), which adversely affects plant photosynthesis. Many studies of PGPR have shown that their application can significantly increase the chlorophyll contents of crops (such as wheat, *Zea mays*, and *Vigna radiate*) grown under saline conditions (Rojas-Tapias et al., 2012; Saghafi et al., 2013; Islam et al., 2016). For examples treatment with *Bacillus amyloliquefaciens* SQR9 increased the total chlorophyll contents (chlorophyll a and b, and carotenoids) in maize (Nadeem et al., 2007; Chen L. et al., 2016). Han et al. (2014) found that the chlorophyll contents of white clover (*Trifolium repens* L. cultivar Huia) increased after inoculation with *Bacillus subtilis* GB03, with a positive effect on plant growth. In the present study, the chlorophyll contents (a, b, and a+b) were also significantly induced by exposure to FZB42 VOCs, which were beneficial for alleviating the negative effects of salt stress on photosynthesis (Figure 2). Furthermore, the high transcription levels of two photosynthesis-related genes (*TPPH* and *LHCB4.3*) induced by VOCs (Figure 3) also confirmed this conclusion.

TSS and proline are often considered as potential biochemical indicators of salt tolerance in plants (Ashraf and Harris, 2004). Many studies have shown that the application of certain types of PGPR is beneficial for the accumulation of TSS and proline in plants against osmotic stress caused by salinity (Upadhyay et al., 2012; Qu et al., 2016). For example, inoculation of *Bacillus amyloliquefaciens* SQR9 enhanced the TSS contents of maize under salt stress condition compared with the non-inoculated control plants (Chen L. et al., 2016). The TSS and proline contents of wheat (Raj-3077) were also significantly increased by *Bacillus subtilis* SU47 and *Arthrobacter* sp. SU18 under salt stress condition (Upadhyay et al., 2012). Similar results were obtained in the present study, as the TSS contents increased significantly after 15 and 20 days of exposure (Figure 5A). On the contrary, the proline contents were significantly lower in the plants exposed to VOCs compared with the non-exposed plants (Figure 5B). Other studies have also shown that PGPR decreased the proline contents of plants. For example, the proline contents of *Arachis hypogaea* shoots inoculated with six PGPR strains were remarkably reduced under salt stress condition (Shukla et al., 2012). Jha et al. (2011) showed that the proline content of rice (*Oryza sativa*) increased as the salt concentration increased, whereas the proline content decreased in rice inoculated with PGPR compared with non-inoculated plants. In addition, *Bacillus amyloliquefaciens* SQR9 reduced the proline contents of maize under salt stress condition (Chen L. et al., 2016). It is possible that the plants treated with PGPR did not experience high salt stress, so the accumulation of proline was low in the presence of PGPR (Shukla et al., 2012).

Reactive oxygen species (ROS) play important roles as signaling molecules in the regulation of numerous biological processes of plants such as plant growth and development, and responses to biotic and abiotic stimuli in plants (Baxter et al., 2014). Besides, high ROS caused by salinity stress can also lead to damage to plant growth by producing oxidative stress (Mittova et al., 2004). ROS can be scavenged and detoxified by enzymatic mechanisms in plants. Antioxidants in plants are considered effective agents that resist oxidative damage under salt stress condition (Spychalla and Desborough, 1990; Mittova et al., 2003). It has been reported that inoculation with PGPR can effectively increase the antioxidant activities in plants, such as the levels of CAT, POS, and SOD, which contribute to salt stress tolerance (Nautiyal et al., 2008; Kasotia et al., 2015; Ullah and Bano, 2015; Islam et al., 2016). In this study, significantly higher levels of antioxidant activities in terms of POD, SOD, and CAT were detected in salt-stressed *Arabidopsis* after exposure to FZB42 VOCs for 20 days compared with the non-exposed plants, but not in those exposed for 15 days (Figure 4). This demonstrated that the FZB42 VOCs could enhance the antioxidant activities and alleviate the oxidative damage caused to plants by salt stress but this effect depended on the exposure time. Carbon dioxide proves to induce an increase in antioxidant activities (Pérez-López et al., 2009). No significant effect of FZB42 VOCs on the antioxidant activities in plants exposed for 15 days was observed, which might be the fact that plants and the zone of FZB42 in plates were small so that the concentration of carbon dioxide was low, further research should be done to confirm this speculation.

It is known that prolonged salt stress leads to ion toxicity due to the increased concentration of Na^+^ and a low K^+^/Na^+^ ratio in plants (Zhang et al., 2010; Kim et al., 2014). Some rhizospheric bacteria can alleviate Na^+^ toxicity in plants by restricting the uptake of Na^+^, extruding Na^+^ into the external environment, sequestering Na^+^ in vacuoles, redirecting Na^+^ from the shoots to the roots, and increasing the K^+^/Na^+^ ratio (Ashraf et al., 2004; Upadhyay et al., 2011; Chen L. et al., 2016). For example, *Bacillus subtilis* (GB03) greatly decreased the accumulation of Na^+^ in the shoots and roots of white clover grown under elevated salt conditions and improved the K^+^/Na^+^ ratio (Han et al., 2014). In whole maize plants, lower Na^+^ contents and higher K^+^/Na^+^ ratios were obtained after inoculation with *Bacillus amyloliquefaciens* SQR9 compared with non-inoculated plants (Chen L. et al., 2016). Similar results were also obtained by Zhang et al. (2008) who reported that the VOCs emitted by *Bacillus subtilis* GB03 facilitated the movement of Na^+^ from the shoots to the roots and restricted the entry of Na^+^ into the roots by down-regulating the expression of *HKT1* in the roots but up-regulating it in the shoots, thereby decreasing the accumulation of Na^+^. In the present study, we also found that FZB42 VOCs induced the expression of *HKT1* in *Arabidopsis* shoots. Moreover, the gene encoding the Na^+^/H^+^ antiporter (*NHX1*) is responsible for Na^+^ sequestration and its overexpression enhances salt tolerance (Apse et al., 1999; Zhang and Blumwald, 2001), and it was also positively regulated by FZB42 VOCs. The up-regulation of these genes might have led to the accumulation of less Na^+^ in plants. Indeed, the Na^+^ contents were significantly lower in the whole plants exposed to FZB42, and higher K^+^/Na^+^ ratios were also obtained in the plants exposed to FZB42 compared with the non-exposed plants (Figure 6). These results are in agreement with those obtained in our previous study where FZB42 was used to inoculate the roots of *Arabidopsis* in a hydroponic system under salt stress (Liu et al., 2017). Together, these results indicate that FZB42 has the capacity to decrease the accumulation of Na^+^ in plants, thereby alleviating the toxicity due to Na^+^ and enhancing the salt tolerance.

It is well known that the phytohormones ABA and JA are involved in the regulation of resistance to abiotic stresses (Delker et al., 2006; Mauch-Mani and Flors, 2009; Tao et al., 2011). But it seems that ABA had no effect on the induction of salt tolerance in plants conferred by FZB42 VOCs, because FZB42 VOCs significantly promoted *abi4-102* growth under salt stress condition (Figure 7), and it also significantly suppressed the expression of *NCED3* that involved in ABA synthesis in plants under salt stress condition (Figure 3). And this result was similar to Chen L. et al. (2016) who found that the expression of *NCED* was also down-regulated with the inoculation of SQR9 under salt stress condition.

Jasmonic acid is an important stress-responsive hormone that can regulate plant responses to abiotic and biotic stresses, as well as plant growth and development (Delker et al., 2006). Some studies have shown that JA can act as a positive regulator of salt tolerance and that it is capable of eliciting a defensive response in plants (Bari and Jones, 2009; Dong et al., 2013; Zhao et al., 2014). In our previous study, we found that JA signaling plays important roles in the enhanced salt tolerance induced in *Arabidopsis* by FZB42 via root colonization (Liu et al., 2017). In the present study, several *Arabidopsis* mutant lines were tested to elucidate the signaling networks involved in the induction of salt tolerance by FZB42 VOCs. The VOCs only failed to promote the growth of the *jar1-1* and *myc2* mutants under salt stress condition, thereby suggesting that FZB42 VOCs might alleviate salt tolerance by mainly regulating JA signaling pathways (Figure 7). In addition, the significantly higher transcriptional levels of JA-mediated defense response genes (*JMT*, *PDF1.3*, *PDF1.2c*, and *PDF1.2*) and JA synthesis gene (*LOX4*) (Figure 3), as well as the fact that FZB42 VOCs did not promote plant growth under treatment with DIECA, further confirmed the role of JA signaling in the induction of salt tolerance conferred by FZB42 VOCs (Figure 8). However, different results were obtained in other studies. Liu et al. (2017) found that in addition to JA signaling, ET signaling also played important roles in mediating systemic salt stress tolerance after inoculating FZB42 on the roots. In addition, Bhattacharyya et al. (2015) showed that VOCs emitted by *Alcaligenes faecalis* JBCS1294 induced salt tolerance in *Arabidopsis* by modulating the auxin and gibberellin pathways. These different results might be explained by the particular inoculation methods employed or differences in chemical signaling by PGPR VOCs (Farag et al., 2006).

In addition, JA signaling plays crucial roles in plant defensive responses against biotic stresses, including herbivores and some microbial pathogens (Browse and Howe, 2008; Bari and Jones, 2009). Studies have shown that the systemic resistance induced in plants by PGPR is also JA-dependent (Pozo et al., 2008). According to our results and those obtained previously, the colonization of *Arabidopsis* roots by the beneficial rhizobacterial strain *Bacillus amyloliquefaciens* FZB42 as well as the volatile emissions from this PGPR induce the expression of JA-responsive genes, thereby leading to induced systemic tolerance and effective protection from salt stress (Liu et al., 2017). Thus, we suggest that JA signaling pathways may play key roles in crosstalk with PGPR to facilitate resistance to both biotic and abiotic stresses, but more research is still needed to support this hypothesis.

In addition, the component of FZB42 VOCs was analyzed by GC-MS, thirty-eight components were identified (Supplementary Figure S6 and Supplementary Table S3). Some of them were also detected in other stains like acetone, 2,3-Butanedione, acetic acid, acetoin, butanoic acid, Pentanone, Decane, Tetramethyl-pyrazine, Hexadecane, Dodecane, 2-Tridecanone, 2-Undecanone, Tridecane (Ryu et al., 2003; Farag et al., 2006; Lee et al., 2012; Bhattacharyya and Lee, 2017; Tahir et al., 2017), and some of them were firstly detected in this study. Acetoin was found the second largest component of FZB42 VOCs, and acetoin of *Bacillus* has reported to be plant growth promoter (Ryu et al., 2003). The results of △*alsD* and △*alsS* on plant biomass were inconsistent with this conclusion, as △*alsD* and △*alsS* still significantly enhanced plant biomass under non-salt and salt tress condition. However, acetoin might have slight effects under salt stress condition, as the fresh weight of △*alsD* and △*alsS* exposed plants were significantly lower than FZB42 exposed plants and significantly higher than non-exposed plants (Supplementary Figure S7). This might be explained by the fact that the components of bacteria VOCs depend on strains, different strains produce different VOCs (Farag et al., 2006), which had different active components to promote plant growth under normal and stress conditions (Zou et al., 2010; Park et al., 2015). Further researches to clarify the active component of FZB42 VOCs will be done in the future.

## Conclusion

In this study, we showed that the VOCs emitted by FZB42 induced salt tolerance in *Arabidopsis* under salt stress by managing Na^+^ homeostasis, maintaining the chlorophyll contents, stabilizing the osmotic potential by increasing the TSS contents, and enhancing the capacity for scavenging ROS by increasing the antioxidant activities of POD, SOD, and CAT. The transcriptional levels of several genes related to photosynthesis, JA synthesis, and especially JA-mediated defense responses were differentially up-regulated by FZB42 VOCs, thereby helping to enhance plant salt tolerance. Mutant analysis also confirmed the important roles of JA signaling in the induction of salt tolerance by exposure to FZB42 under salt stress condition. Our findings provide new insights into the induction of salt tolerance conferred by PGPR but further research is required to determine the effects of individual VOCs from FZB42 on induced systemic tolerance to salt stress.

## Data Availability Statement

The raw data supporting the conclusions of this article will be made available by the authors, without undue reservation.

## Author Contributions

SL and RW conceived and designed the experiments. SL and YT performed the experiments and wrote the manuscript. SL, MJ, XL, and LY analyzed the data. RW, XZ, ZX, and WJ provided the technical assistance to SL. YW and YZ revised the language of the article. All the authors contributed to the article and approved the submitted version.

## Conflict of Interest

The authors declare that the research was conducted in the absence of any commercial or financial relationships that could be construed as a potential conflict of interest.

## Supplementary Material

Supplementary Figure 1 Effect of FZB42 VOCs on the growth of WT roots. **(A,B)** Indicated the phenotype of roots in plates containing water and root length. White, light gray, dark gray, and black bars represent CK (only water), FZB42 VOCs (only FZB42 VOCs), Salt (only salt stress), and Salt + FZB42 VOCs (salt stress + FZB42 VOCs) treatments, respectively. Different letters indicate statistically significant differences between treatments (Duncan’s multiple range tests, *P* < 0.05; *n* = 40, mean ± standard deviation).

Supplementary Figure 2 Effects of DH5α VOCs on growth and salt tolerance in *Arabidopsis*. Representative images of *Arabidopsis* after exposure to VOCs from DH5α or water for 15 and 20 days, respectively **(A,B)**. Effects of DH5α VOCs on the shoot fresh weight **(C)**, shoot dry weight **(D)**, root fresh weight **(E)**, and lateral root number **(F)**. White, light gray, dark gray, and black bars represent CK (only water), DH5α VOCs (only DH5α VOCs), Salt (only salt stress), and Salt + DH5α VOCs (salt stress + DH5α VOCs) treatments respectively. Different letters indicate statistically significant differences between treatments (Duncan’s multiple range tests, *P* < 0.05; *n* = 40, mean ± standard deviation).

Supplementary Figure 3 Effects of FZB42 VOCs on growth and salt tolerance in *Arabidopsis* mutants.

Supplementary Figure 4 Effects of FZB42 VOCs on the growth phenotype of *Arabidopsis* mutant roots under non-salt and salt stress conditions.

Supplementary Figure 5 Effects of FZB42 VOCs on the root length of *Arabidopsis* mutants under non-salt and salt stress conditions. White, light gray, dark gray, and black bars represent CK (only water), FZB42 VOCs (only FZB42 VOCs), Salt (only salt stress), and Salt + FZB42 VOCs (salt stress + FZB42 VOCs) treatments respectively. Different letters indicate statistically significant differences between treatments (Duncan’s multiple range tests, *P* < 0.05; *n* = 30, mean ± standard deviation).

Supplementary Figure 6 SPME-GC/MS chromatogram profiles of VOCs emitted from FZB42. Compounds identified positively as (1) Acetone, (2) 2,3-Butanedione, (3) 3-methyl-2-Butanone, (4) Acetic acid, (5) acetoin, (6) 3-methyl-2-Pentanone, (7) 3,4,5-Trimethylpyrazole, (8) Hexamethyl-cyclotrisiloxane, (9) Butanoic acid, (10) ethylbenzene, (11) *p*-xylene, (12) Styrene, (13) 2-Heptanone, (14) Nonane, (15) 2-Pentanone, (16) 5-methyl-2-heptanone, (17) Decane, (18) octamethyl-cyclotetrasiloxane, (19) 2-Dodecanone, (20) Tetramethyl-pyrazine, (21) 2-Nonanone, (22) Hexadecane, (23) 5-methoxy-thiazole, (24) 2-Decanone, (25) Naphthalene, (26) Dodecane, (27) 2-Tridecanone, (28) 2-methyl-Naphthalene, (29) 2-Undecanone, (30) Tridecane, (31) 2-methyl-tetradecane, (32) Tetradecane, (33) Pentadecane, (34) Tetradecamethyl-cycloheptasiloxane, (35) 2-Tetradecanone, (36) Hexadecamethyl-cyclooctasiloxane, (37) Heptadecane, (38) Octadecamethyl-cyclononasiloxane.

Supplementary Figure 7 Effects of △*alsD* and △*alsS* on plant growth under non-salt and salt stress conditions. **(A–D)** Indicated the representative images of water, FZB42, △*alsD*, and △*alsS* exposed *Arabidopsis* under non-salt stress condition, respectively. **(F–I)** Indicated the representative images of water, FZB42, △*alsD*, and △*alsS* exposed *Arabidopsis* under salt stress condition, respectively. **(E,J)** Indicated the fresh weight of plants under non-salt and salt stress conditions, respectively.

Supplementary Figure 8 Growth curve of FZB42, △*alsD* and △*alsS*.

Supplementary Table 1 qRT-PCR primer sequences used in this study.

Supplementary Table 2 Original data of this study.

Supplementary Table 3 VOCs profile of *Bacillus amyloliquefaciens* FZB42.
