# Supplementary figures and images for: Induction of Salt Tolerance in Arabidopsis thaliana by Volatiles From Bacillus amyloliquefaciens FZB42 via the Jasmonic Acid Signaling Pathway
Source: Front Microbiol. 2020 Nov 12;11:562934. doi: 10.3389/fmicb.2020.562934 (PMC7688926; doi:10.3389/fmicb.2020.562934)

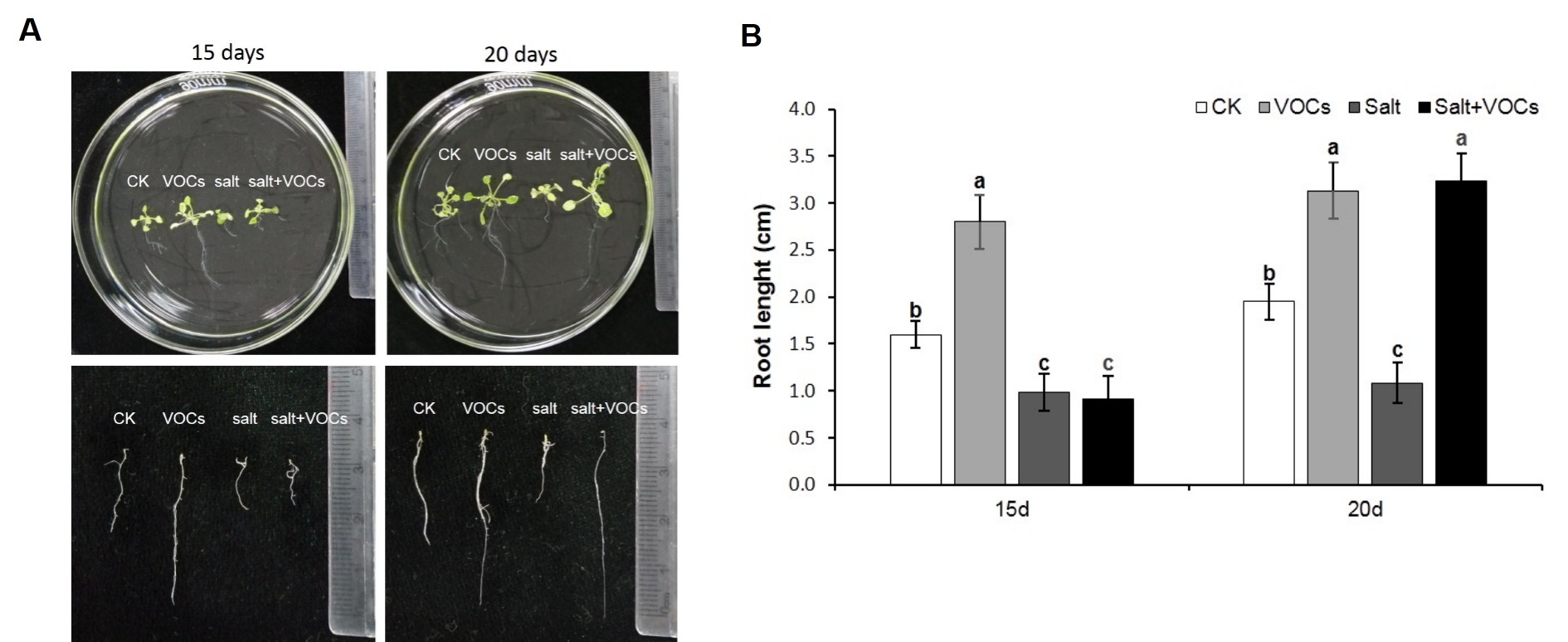

Supplement: Supplementary Figure 1 — Effect of FZB42 VOCs on the growth of WT roots. (A,B) Indicated the phenotype of roots in plates containing water and root length. White, light gray, dark gray, and black bars represent CK (only water), FZB42 VOCs (only FZB42 VOCs), Salt (only salt stress), and Salt + FZB42 VOCs (salt stress + FZB42 VOCs) treatments, respectively. Different letters indicate statistically significant differences between treatments (Duncan’s multiple range tests, P < 0.05; n = 40, mean ± standard deviation). [file Image_1.jpeg]

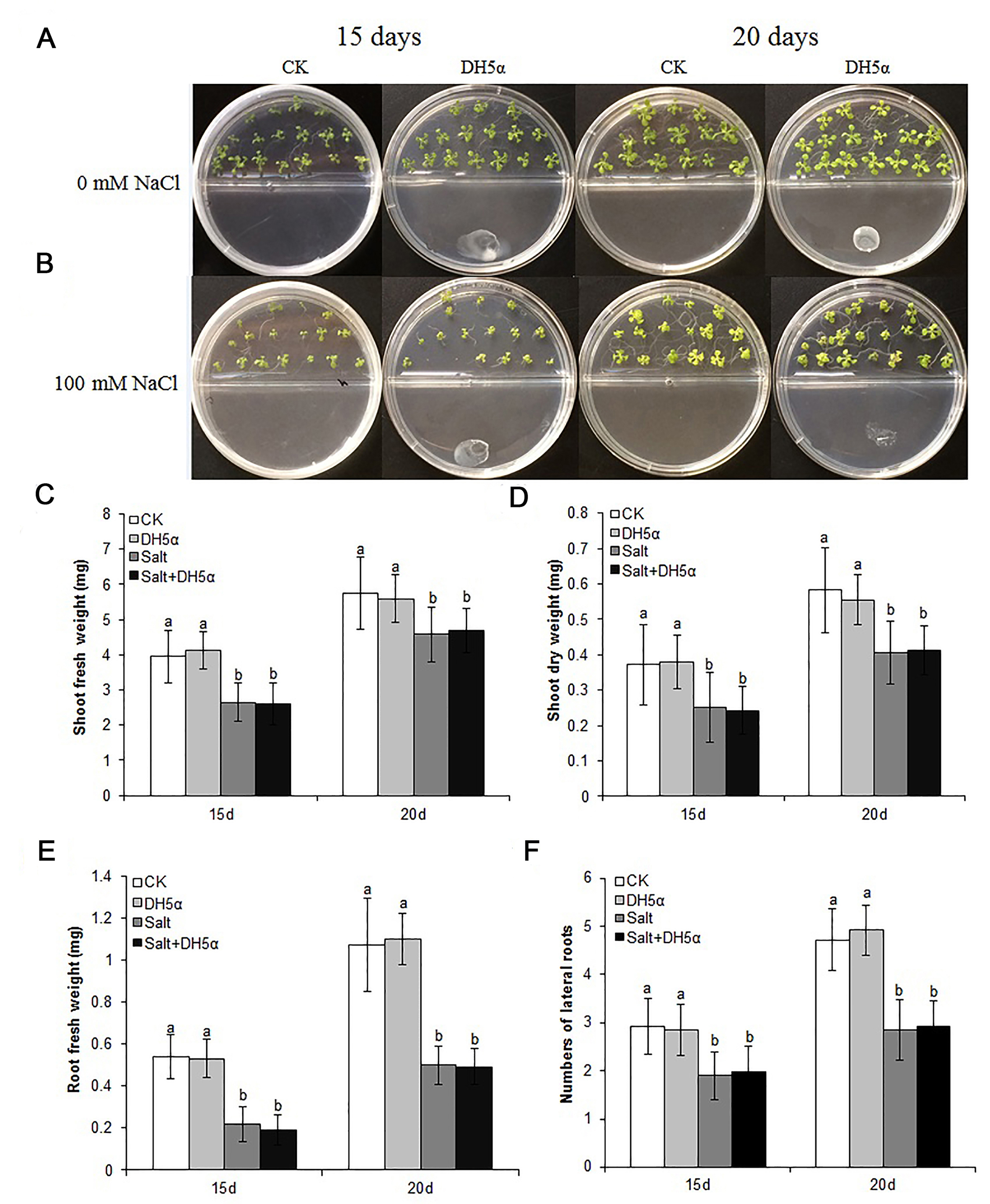

Supplement: Supplementary Figure 2 — Effects of DH5α VOCs on growth and salt tolerance in Arabidopsis. Representative images of Arabidopsis after exposure to VOCs from DH5α or water for 15 and 20 days, respectively (A,B). Effects of DH5α VOCs on the shoot fresh weight (C), shoot dry weight (D), root fresh weight (E), and lateral root number (F). White, light gray, dark gray, and black bars represent CK (only water), DH5α VOCs (only DH5α VOCs), Salt (only salt stress), and Salt + DH5α VOCs (salt stress + DH5α VOCs) treatments respectively. Different letters indicate statistically significant differences between treatments (Duncan’s multiple range tests, P < 0.05; n = 40, mean ± standard deviation). [file Image_2.jpeg]

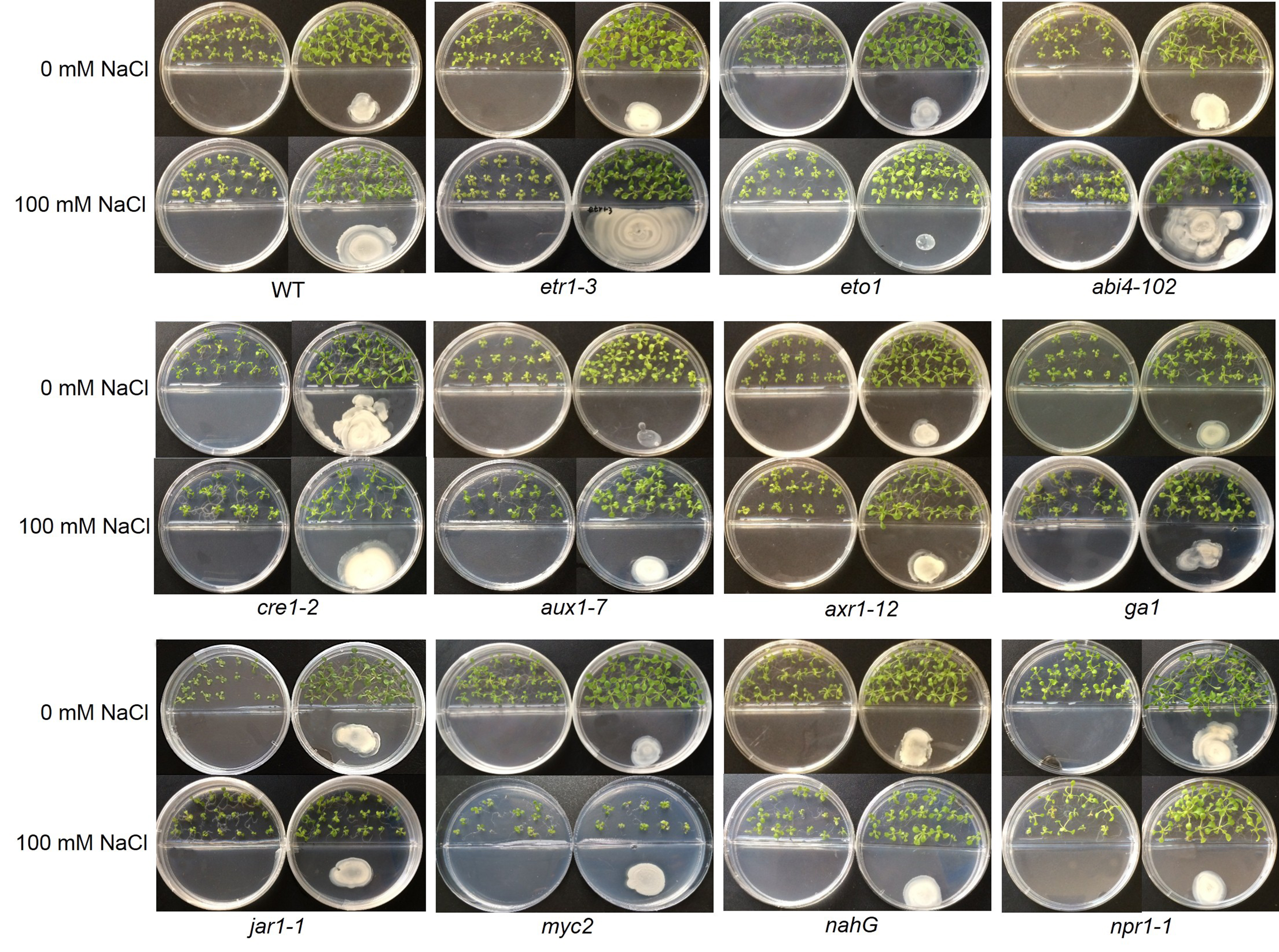

Supplement: Supplementary Figure 3 — Effects of FZB42 VOCs on growth and salt tolerance in Arabidopsis mutants. [file Image_3.jpeg]

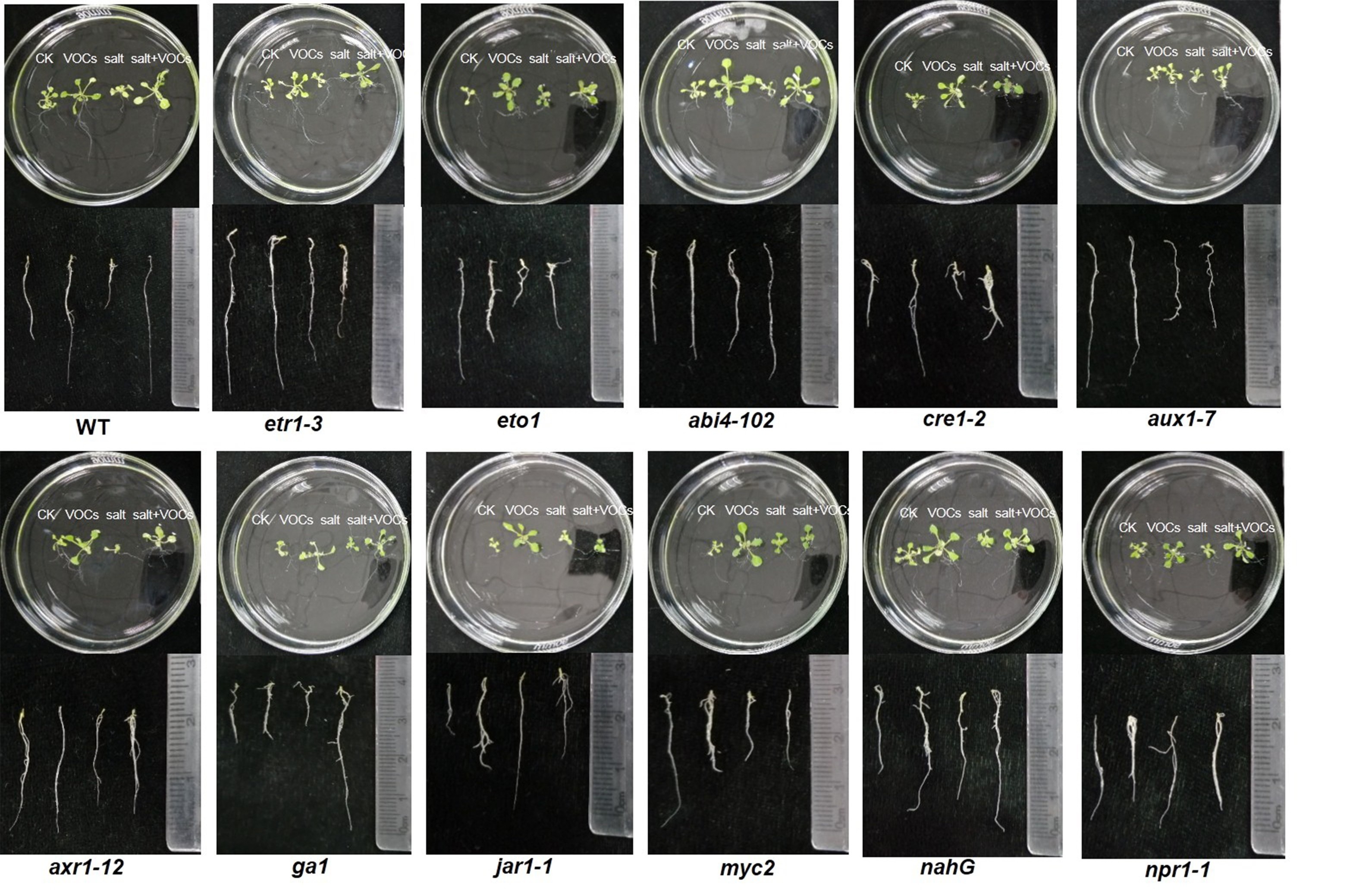

Supplement: Supplementary Figure 4 — Effects of FZB42 VOCs on the growth phenotype of Arabidopsis mutant roots under non-salt and salt stress conditions. [file Image_4.jpeg]

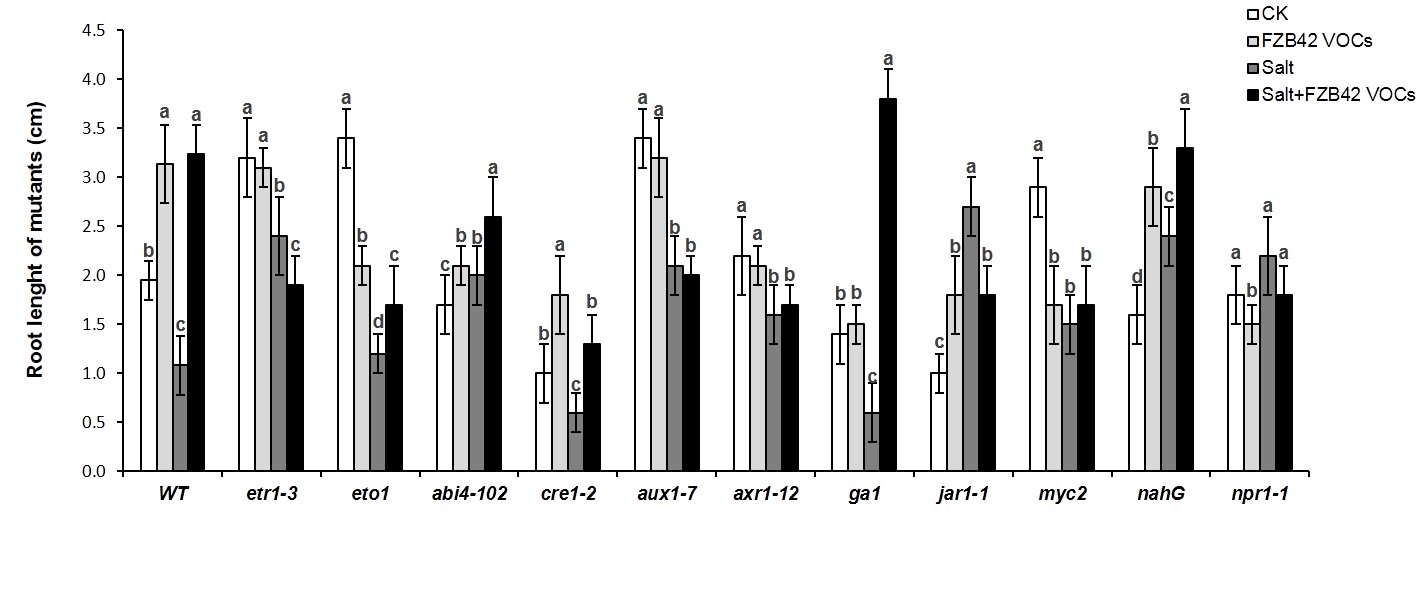

Supplement: Supplementary Figure 5 — Effects of FZB42 VOCs on the root length of Arabidopsis mutants under non-salt and salt stress conditions. White, light gray, dark gray, and black bars represent CK (only water), FZB42 VOCs (only FZB42 VOCs), Salt (only salt stress), and Salt + FZB42 VOCs (salt stress + FZB42 VOCs) treatments respectively. Different letters indicate statistically significant differences between treatments (Duncan’s multiple range tests, P < 0.05; n = 30, mean ± standard deviation). [file Image_5.jpeg]

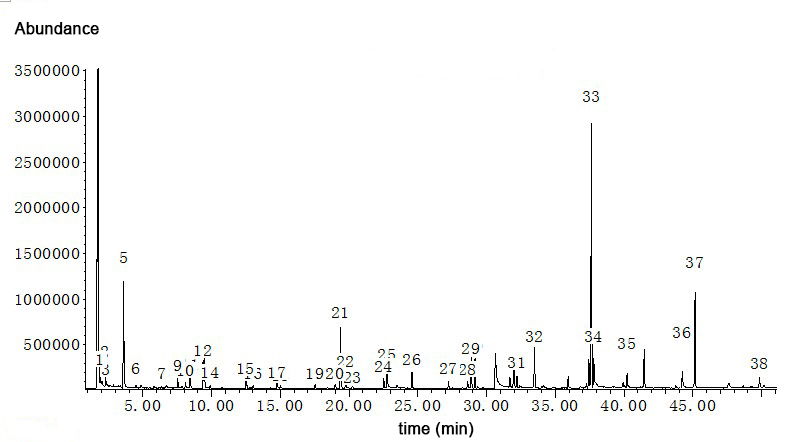

Supplement: Supplementary Figure 6 — SPME-GC/MS chromatogram profiles of VOCs emitted from FZB42. Compounds identified positively as (1) Acetone, (2) 2,3-Butanedione, (3) 3-methyl-2-Butanone, (4) Acetic acid, (5) acetoin, (6) 3-methyl-2-Pentanone, (7) 3,4,5-Trimethylpyrazole, (8) Hexamethyl-cyclotrisiloxane, (9) Butanoic acid, (10) ethylbenzene, (11) p-xylene, (12) Styrene, (13) 2-Heptanone, (14) Nonane, (15) 2-Pentanone, (16) 5-methyl-2-heptanone, (17) Decane, (18) octamethyl-cyclotetrasiloxane, (19) 2-Dodecanone, (20) Tetramethyl-pyrazine, (21) 2-Nonanone, (22) Hexadecane, (23) 5-methoxy-thiazole, (24) 2-Decanone, (25) Naphthalene, (26) Dodecane, (27) 2-Tridecanone, (28) 2-methyl-Naphthalene, (29) 2-Undecanone, (30) Tridecane, (31) 2-methyl-tetradecane, (32) Tetradecane, (33) Pentadecane, (34) Tetradecamethyl-cycloheptasiloxane, (35) 2-Tetradecanone, (36) Hexadecamethyl-cyclooctasiloxane, (37) Heptadecane, (38) Octadecamethyl-cyclononasiloxane. [file Image_6.jpeg]

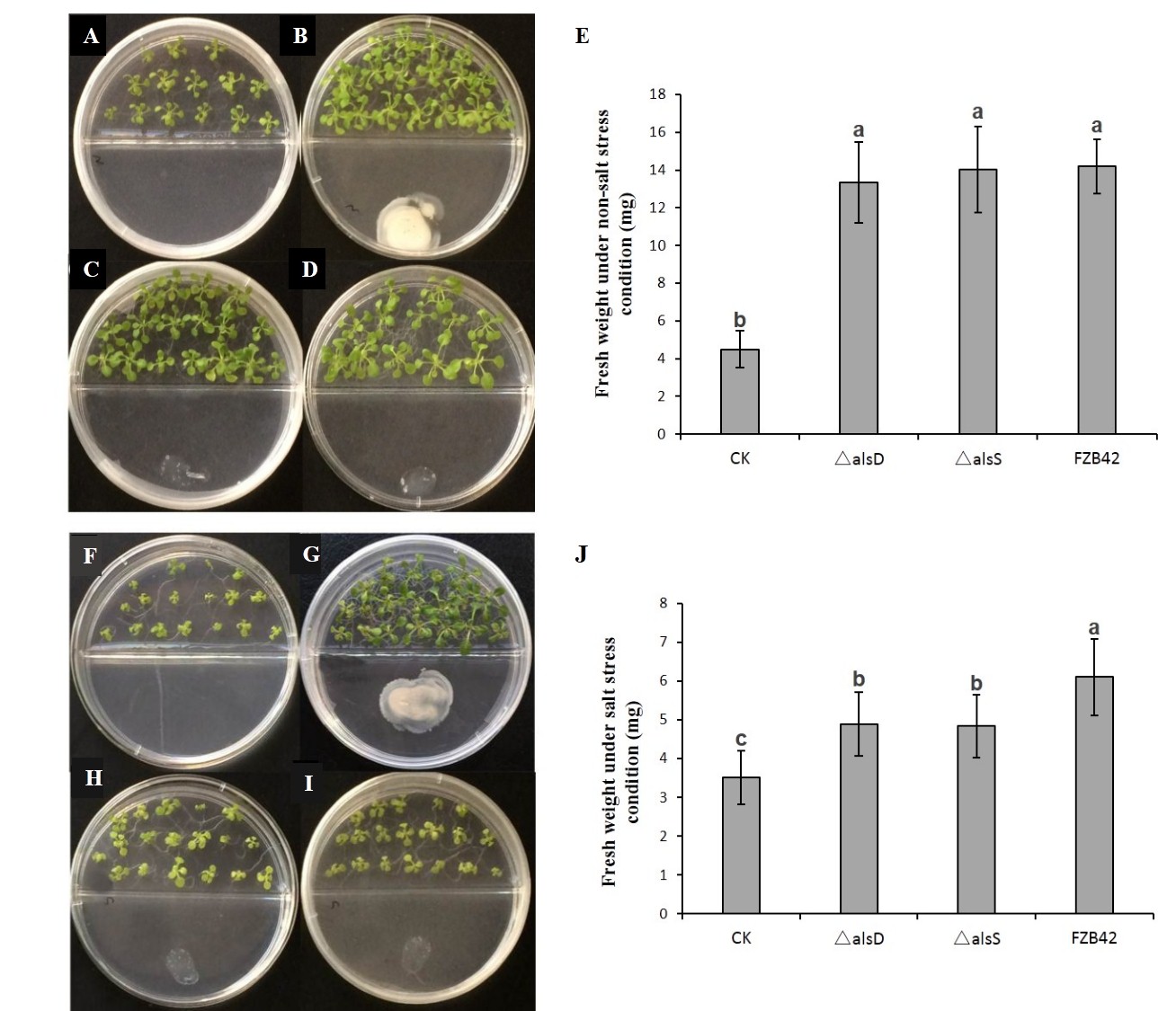

Supplement: Supplementary Figure 7 — Effects of △alsD and △alsS on plant growth under non-salt and salt stress conditions. (A–D) Indicated the representative images of water, FZB42, △alsD, and △alsS exposed Arabidopsis under non-salt stress condition, respectively. (F–I) Indicated the representative images of water, FZB42, △alsD, and △alsS exposed Arabidopsis under salt stress condition, respectively. (E,J) Indicated the fresh weight of plants under non-salt and salt stress conditions, respectively. [file Image_7.jpeg]

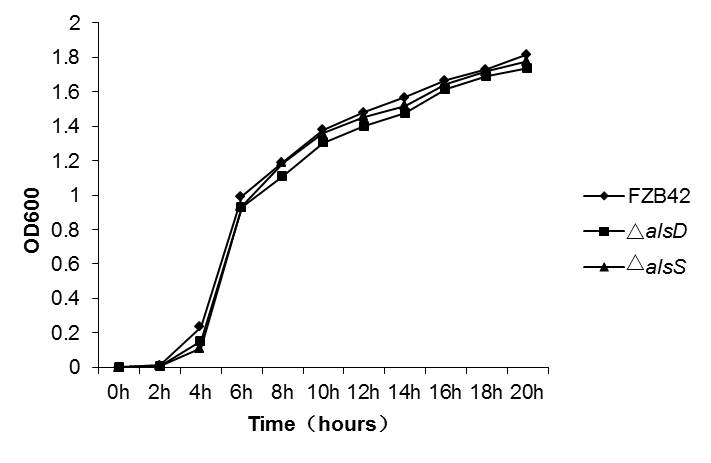

Supplement: Supplementary Figure 8 — Growth curve of FZB42, △alsD and △alsS. [file Image_8.jpeg]
